# The role of chest ultrasonography in the management of respiratory diseases: document II

**DOI:** 10.1186/2049-6958-8-55

**Published:** 2013-08-09

**Authors:** Andrea Smargiassi, Riccardo Inchingolo, Gino Soldati, Roberto Copetti, Giampietro Marchetti, Alessandro Zanforlin, Rosangela Giannuzzi, Americo Testa, Stefano Nardini, Salvatore Valente

**Affiliations:** 1 Pulmonary Medicine Department, Università Cattolica del Sacro Cuore, University Hospital “A. Gemelli”, Roma, Italy; 2 Emergency Department, General Hospital “ASL 2 Valle del Serchio”, Castelnuovo di Garfagnana, Lucca, Italy; 3 Emergency Department, GeneralHospital “ASS 5 BassaFriulana”, Latisana, Udine, Italy; 4 Pulmonary Medicine Department, “Spedali Civili”, Brescia, Italy; 5 Pulmonary Medicine Unit, General Hospital, Trecenta, Rovigo, Italy; 6 Emergency Department, Università Cattolica del Sacro Cuore, University Hospital “A. Gemelli”, Roma, Italy; 7 Internal Medicine Unit, Private Hospital “Madonna delle Grazie”, Velletri, Roma, Italy; 8 Pulmonary and TB Unit, Vittorio Veneto General Hospital, Vittorio Veneto, TV, Italy

**Keywords:** Chest ultrasonography, Interventional pulmonology, Pulmonary consolidations, Pulmonary edema, Pulmonary embolism, Sonographic interstitial syndrome

## Abstract

Chest ultrasonography can be a useful diagnostic tool for respiratory physicians. It can be used to complete and widen the general objective examination also in emergency situations, at the patient’s bedside. The aim of this document is to promote better knowledge and more widespread use of thoracic ultrasound among respiratory physicians in Italy.

This document II is focused on advanced approaches to chest ultrasonography especially in diagnosing sonographic interstitial syndrome with physical hypotheses about the genesis of vertical artifacts, differential diagnosis of cardiogenic pulmonary edema and non-cardiogenic pulmonary edema, raising diagnostic suspicion of pulmonary embolism, ultrasound characterization of lung consolidations and the use of ultrasonography to guide procedural interventions in pulmonology.

Finally, document II focuses on chest ultrasonography as useful diagnostic tool in neonatal and pediatric care.

## Review

### Introduction

For many years transthoracic ultrasound was limited exclusively to the examination of pleural effusions. However, over the past few years ultrasonography of the pleural space and lung parenchyma is gaining a wide consensus in different conditions in clinical practice, particularly in emergency conditions. The aim is to integrate the lung ultrasound information with the echocardiographic information - well-established - on the cardiovascular system. Given its ease of use and bed-side practicality and its simple, linear semiotics, pleural–parenchymal lung ultrasound can and must offer a useful tool for respiratory specialists.

Despite its characteristics, use of thoracic ultrasonography has gained a wider ground in emergency medicine and intensive care than in respiratory divisions. However, with the increasing implementation of Respiratory Intensive Care Units (RICU) and of Semi–Intensive Respiratory Units under the management of respiratory specialists, the need now has become urgent to raise awareness about this critical issue among respiratory specialists so that they too embrace this method.

Respiratory specialists, in fact, deal with situations of critical care and manage patients with high levels of care complexity in whom lung ultrasound represents a useful diagnostic tool. Integrated lung ultrasound - understood as a multiregional, multi-probe bedside examination aimed at answering a few simple queries (yes/no response) that have emerged from the clinical evaluation (medical history and objective examination), i.e. integrating the ultrasonographic and clinical data - has been found to be of extreme help in the clinical practice of respiratory care. In addition, its use can also be considered of great interest as a diagnostic tool in elective conditions in the examination of many pleural-parenchymal diseases and in monitoring their follow up.

The main aspects of chest ultrasonography dealt with in this Document II, can be identified as follows:

1) Differential diagnosis in “sonographic interstitial syndrome”.

2) Differential diagnosis of cardiogenic and noncardiogenic acute pulmonary edema.

3) Raising diagnostic suspicion of pulmonary embolism.

4) Ultrasound characterization of lung consolidations.

5) Ultrasound-guided interventional procedures.

6) Lung ultrasound in neonatal and pediatric care.

### Differential diagnosis in “sonographic interstitial syndrome”

The underlying principle on which B-mode ultrasound is based is the possibility of ultrasounds to penetrate structures with similar acoustic impedance and for morphologic anatomic images to be constructed from the interpretation of the “echoes” that return to the transducer. This is the main reason why the application of ultrasound to the study of chest structures has always been limited only to the examination of pleural effusions and cardiovascular structures. But it is becoming increasingly useful in clinical practice also to study the role of ultrasounds in the case of interference with ventilated lung, where the marked difference of acoustic impedance between air and lung tissue/liquids impedes the penetration of the ultrasounds.

The property that allows the propagation of the sound waves in the medium is a function of the acoustic impedance (Z) of the medium itself and is expressed in rayls; for a flat wave:

Z=ρc

where ρ is the density of the medium and c the velocity of propagation in the medium. When the ultrasonic beam crosses different adjacent mediums with diverse impedance the reflection of the ultrasound wave is explained by the following relation where R is the coefficient of reflection and Z^1^ and Z^2^ the impedances of the different contiguous mediums:

$$R=\left(Z^{2}-Z^{1}\right)/\left(Z^{2}+Z^{1}\right)$$

The chest wall and ventilated lung behave at ultrasound examination in a very different manner in that the acoustic impedances of the two systems are quite different. Consequently the pleural line behaves as a reflecting mirror of the ultrasounds that have crossed the layers above. It can be derived from this that pleural–parenchymal lung ultrasound examination cannot be morphological, but artifactual and dynamic.

Sonographic interstitial syndrome stands for a particular lung condition in which the impact of the acoustic waves on the first pleural–parenchymal layers does not cause a simple “mirror-like” reflection, but rather a series of acoustic phenomena that manifest themselves, in B-mode scanning, in the production of vertical artifacts defined as B lines.

The presence and/or confluence of these vertical artifacts defines a “break” of the mirror and the presence of sonographic interstitial syndrome [1].

It is essential thus to understand what a vertical artifacts is. The original description referred to an artifact visually analogous to the “ring down”, first called “comet tail artifact”. Its description was purely qualitative: “artifact due to reverberation, hyperechogenic, with a narrow point of origin that expands like a laser beam to the (lower) margin of the screen” [2]. The latest hypothesis on the physical phenomenon indicates that a B line could be produced when the ultrasound beam can see, in relation to its wavelength, a non impedant structure (a sort of acoustic micro hole), that can allow it to infiltrate highly reflective surfaces that can provoke focal reverberations, modified by eventual interferences, that the machine reads as a close succession in depth of echoes [1].

B lines in lung ultrasonography have diverse concentration and homogeneity at the pleural level. Their coalescence makes the image assume a characteristic type of echogenicity, defined rather generically as “white lung” (completely white sonographic lung field with or without B lines emerging and without horizontal artifacts due to reverberation”), but it has not in fact been demonstrated if the homogeneous echogenicity of certain areas of white lung represents really a coalescence of B lines or a different phenomenon. The definition of B lines has remained the same for many years in that their physical origin remains unknown.

We know without doubt that they are artifacts: a B line cannot represent any anatomic structure of the lung because normal or pathologic lung does not have structures that are vertical, orthogonal at pleural level, linear and extended for the whole length such as B lines have; moreover images similar to B lines can be reproduced *in vitro* scanning various substrates (such as foam or porous “soaked” polyurethane) [3] in which the structural linearity is not present. Also what goes under the name of “white lung” probably represents an artifact of amplification in that it masks any real structure eventually present beneath it, and this is manifested both *in vivo* and *in vitro*.

Also from the methodological point of view B lines have been evaluated in diverse studies with different probes and frequencies. In this way what appears with one type of probe is not the same as what is manifested using another type. Also the frequency and inclination of the probe are important variables in the observation of these phenomena [4].

A linear probe utilizing higher frequencies in the presence of interstitial syndrome shows more artifacts and, in particular, allows to observe with greater detail the presence of different types of artifact: it allows to distinguish between long artifacts (the whole screen) and short ones (1 cm or less), and often to clarify the origin of the artifact (in the form of a point or in certain cases micronodular), allows a more precise definition of their surface confluence and of white lung [4].

Previous studies on bioacoustics [5,6], the possibility of reproducing B lines on different substrates such as porous polyurethane soaked in water and the evidence that they increase with the increased level of soaking and thus reduction of porosity, have nonetheless favored the development of new fields of research. In fact it has been demonstrated [7] that also healthy lung can produce such vertical artifacts if artificially “deflated” to a level not physiologically attainable. It is, hence, the reduction of porosity or increased density of the lung parenchyma that can cause an acoustic interruption of the pleural line.

The production of B lines can then signal a variation of density, i.e. less porosity of the lung, considering the peripheral lung parenchyma similar to the structure of a foam.

The limit of B line production (and of “white lung”) is the critical density that generates consolidation. Pure consolidation has a density very close to 1 g/ml. Healthy lung physiologically ventilated has a density of 0.15 g/ml. Parenchymal lung disease which provokes an increase of lung density (decrease of lung porosity) - whether it makes the lung lose air or increases its solid (water/tissue) content, or does both - favors the production of different concentrations of B lines, before the tissue reaches the critical density for expressing images of consolidation.

There exist in reality several types of possible “generators” of vertical artifacts:

1) single or multiple interlobular and/or intralobular septa thickening,

2) increase of lung parenchymal water content (increased density),

3) increase of “tissue” content of the lung parenchyma (increased density),

4) diminution of ventilation and porosity due to any cause (increased density),

5) micronodules, pleural irregularities and subpleural microconsolidations.

Each of these conditions constitutes an alteration that can “break” the mirror of the pleural line and cause sonographic vertical artifact phenomena. In some cases it is possible to easily trace the cause to a unifying hypothesis (increased density); in other cases it is a more difficult exercise. In effect, and also experimentally, it is possible to observe that not all vertical artifacts are equal and behave in the same way. There exists, therefore, a concrete limit in the overall, general definition of these artifacts as “B lines”.

These differences in vertical artifacts appear increasingly important in the description of interstitial syndromes and, above all, probably there can be diverse phenomena present “contemporaneously” that together describe the sonographic interstitial pattern, without one theory on the physical genesis necessarily contrasting and completely confuting another one. Whatever the origin is, the study of vertical artifacts is widely used in clinical practice as an index of lung pathology [8-10].

These artifacts possess an excellent specificity and sensitivity in intercepting the cases of lung pathology. They are associated with practically absolute sensitivity with radiologic (CT) patterns of diffuse infiltrative lung disease, ground glass and subpleuralmicronodules.

Nonetheless, it should be stressed that in reality they are only “errors” of ultrasound machines that interpret “in their own manner” acoustic interactions. In reality we currently are able just to roughly sketch a theory concerning the genesis of these artifacts and we do not know what the best frequency or the best probe is to detect them, or what the best device is for making them appear in a mode that is unequivocal [4].

Concerning the distinctive characteristics of sonographic interstitial syndromes, they can be classified on the basis of their extent, as: focal or diffuse.

Focal sonographic interstitial syndrome is topographically detectable only in relation to limited zones of pleural-parenchymal pathological alterations. This condition can be observed in:

– focal interstitial pneumonias,

– areas of impaired ventilation (dysventilation),

– localized inflammation such as in pulmonary infarction and pulmonary contusions,

– areas with altered pleural–parenchymal density close to inflammatory consolidations (pneumonia) and/or consolidations of other nature (neoplasia, atelectasis, etc.…),

– other pleural–parenchymal alterations of moderate extent (scar outcomes, localized fibrotic areas, etc.…).

On the other hand, in cases with a diffuse, bilateral extension it is necessary distinguish whether the vertical artifacts have a homogeneous or dishomogeneous (i.e. with spared areas) appearance.

To the study of the vertical artifacts’ ultrasound distribution must be associated the assessment of the pleural line, which can appear smooth, thin and regular, or thickened, coarse and irregular.

We can thus distinguish two patterns of combination:

– diffuse homogeneous sonographic interstitial syndrome, with a smooth, thin, regular pleural line (pattern A);

– diffuse dishomogeneoussonographic interstitial syndrome, with a thickened, coarse, irregular pleural line (pattern B).

Finally, for an ulterior classification of these diffuse alterations and a further clue to arrive at the presumed diagnosis, one should also consider their gradient of concentration in the apical-caudal or ventral-dorsal sense. Hence a type A pattern involving mostly the caudal and dorsal regions for reasons of gravity can be indicative of cardiogenic pulmonary edema [8,11,12]. A type B pattern with an unclear apical-caudal or ventral-dorsal gradient, which can be associated with multiple subpleural consolidations and clearly spared areas, can be indicative of noncardiogenic pulmonary edema [8,11,13]. A type B pattern with a clear prevalence in the basal regions where the spared areas may be less and with a prevalence of subpleural micro consolidations and extremely irregular, thickened (“cobbled”) pleural line can be indicative of fibrosing diffuse infiltrative lung disease [8,14].

If honeycombing is present there can be present some spared areas with a pseudo normal pattern (presence of horizontal-reverberant artifacts considering the air content of the cystic bullous lesions) or the presence of rough vertical artifacts originating from the tetrahedral disposition of the cystic bullous lesions.

Clearly each suspected diagnosis should be investigated integrating all the information from the pleural–pulmonary ultrasound with that coming from the echocardiography and from the objective clinical assessments.

### Differential diagnosis of cardiogenic and noncardiogenic acute pulmonary edema

Cardiogenic acute pulmonary edema (APE) and noncardiogenic acute pulmonary edema (found in acute lung damage, acute lung injury [ALI], and acute respiratory distress syndrome [ARDS]) constitute two typical examples of sonographic interstitial syndrome, clinical realities that the respiratory specialist has to face daily.

Both are characterized by the sign, at lung ultrasound, of confluent, multiple B lines up to the characteristic expression of “white lung”. An ultrasound pattern of white lung delineates the presence of pulmonary edema with 100% sensitivity; vice versa, an ultrasound pattern of normality excludes the possibility of an acute pulmonary edema. The concentration of B lines is variable, as also is their diffusion in the lung fields which can be influenced, at least in part, by gravity.

The most common method of imaging used today for detecting acute pulmonary edema is chest radiography. This method, in an acute setting, is at times difficult to interpret and has a certain inter-observer variability [15]. Moreover, increased values of pulmonary capillary wedge pressure (PCWP) do not necessarily produce radiographic data indicative of APE [16]. The gold standard for evidencing pulmonary congestion is the measuring of PCWP by catheterization, a procedure not carried out routinely in respiratory units, even intensive ones. Hence the importance of methods that can reveal signs of pulmonary edema instantly “at the patient’s bedside” such as lung ultrasound, integrating the dynamic estimates it provides with the clinical assessment of the patient.

It has been established the correlation between presence and distribution of B lines and: i) Kerley B lines that can be evidenced with chest X-ray [17], ii) volume of extravascular lung water, measured invasively by thermo dilution method [12], and iii) severity of diastolic dysfunction [18] in patients with APE.

In patients with decompensated heart failure (systolic and/or diastolic), decompensation expressed in terms of acute pulmonary edema, lung sonography can allow to evidence: 1) direct signs of edema, 2) pleural effusions, particularly useful for diagnosis, especially if bilateral, 3) eventual consensual areas of atelectasis, consolidations, masses or raised hemidiaphragm (this can be carried out at the patient’s bedside with serial controls integrated with clinical assessment) [19,20], 4) evaluation of the caval index and estimate of central venous pressure [21].

Particularly useful for respiratory specialists is the use of lung ultrasonography to differentiate between cardiogenic and noncardiogenic acute pulmonary edema. This distinction is significant especially on account of the high mortality that characterizes ARDS [22]. In this clinical context, lung ultrasound shows a sensitivity and specificity of 98% and 88%, respectively, in early detection of pulmonary edema that characterizes the clinical syndrome [13]. Being a condition characterized by pulmonary edema, ARDS presents a sonographic pattern of interstitial syndrome with multiple dense B lines leading to a pattern of white lung, similar in some aspects to what is observed in APE. Some proprieties of this interstitial syndrome enable, anyway, a clear distinction from cardiogenic acute pulmonary edema. In particular, ARDS manifests alterations of the pleural line, in terms of irregular thickening and small subpleural consolidations and “spared areas”, i.e. zones with a normal ultrasound aspect interposed with areas of interstitial syndrome signs varying from multiple, dense B lines to pulmonary consolidations up to white lung [11].

Finally, the possibility to integrate information from lung ultrasonography with echocardiography may allow a complete definition and distinction of cardiogenic and non-cardiogenic acute pulmonary edema.

### Raising diagnostic suspicion of pulmonary embolism

Pulmonary embolism in itself does not in the acute phase cause lung parenchymal alterations that are visible at lung ultrasound. Nonetheless, there are some indirect signs that can be observed, thanks in particular to the aid of integrated cardiovascular and lung sonography. Thus a multidirectional strategy is delineated that can be defined as “killing three birds with one stone”, by which a scan of the lung surfaces should be followed by visualization of the right heart chambers and then by assessment of the femoral-popliteal veins in the search for deep vein thrombosis (DVT) [4].

It is clear that the suspicion of pulmonary embolism should be raised in all cases of acute dyspnea with a normal pleural-pulmonary ultrasound picture and in the presence of risk factors.

Some sonographic signs, however, can sometimes be found. Pulmonary embolism in fact can be associated to infarct lesions with a frequency reported in the anatomo-pathologic literature of 22% [23]. However, pulmonary embolism is a highly dynamic process and it is known that subpleural consolidations occur with high frequency in CT studies and not always do they appear as infarcts but as regions of atelectasis or with exudate [24].

Sonographically lesions have been described, if recent, as hypo-echogenic, moderately homogeneous areas, shaped like a rounded wedge, on average of small dimension (excepting in the case of huge infarcts) with a blurred contour or well-defined with respect to the adjacent parenchyma [25-27]. If lesions are smaller than 5 mm it is difficult to distinguish them from fibrotic nodules or pleural scars. The amount of air contained in these consolidations can be very low due to the hypoxic constriction of the afferent bronchus and/or its compression by the exudate and/or the alveolar collapse following surfactant loss and extravasation of liquids and erythrocytes. It is rare to see a clear air bronchogram in a recent embolism. Infarcts less recent appear better demarcated, clearly wedge-shaped and with central echoes corresponding to the bronchiole as a testimony to their segmental nature. Often (50-60%) there is a thin layer of pleural effusion, invisible at X-ray, in the basal regions or a localized enlargement of the pleural space at the lesion site, due to a “warning-signal” accumulation of liquid [25].

These pleural–parenchymal lung evaluations, as previously said, must be combined and integrated with cardiovascular assessments. Nonetheless, it should be clear that integrated ultrasound can be a highly valid tool for raising the diagnostic suspicion of pulmonary embolism, but it should not be considered a substitute of the established diagnostic procedure: pulmonary angio CT scan remains the gold standard [28].

Concerning the cardiac evaluation, pulmonary embolism produces a variable degree of volume and pressure overload on the right ventricle that can manifest itself through the flattening or the protrusion of the septum towards the left ventricle, respectively in diastole or during the whole cardiac cycle, very evident in parasternal axis view [28].

In addition, the availability of Doppler equipment can allow to estimate right ventricular systolic pressure through the calculation of the tricuspid regurgitant jet velocity in the absence of pulmonary valve stenosis (PAPs estimate). An indirect sign of increased central venous pressure comes also from an evaluation of the inferior vena cava, which typically appears relaxed and non collapsible during the act of breathing. To complete the ultrasound diagnostic process it is necessary to carry out also an assessment of the large veins of the lower limbs through venous compression ultrasound (CUS) and if available through color Doppler assessment. Deep vein thrombosis (DVT) of the lower limbs is generally the cause of pulmonary embolism. A diagnosis of DVT, in a person with compatible symptoms, is highly predictive of pulmonary embolism.

CUS of the lower limbs is indicated in the case of abnormal values of D-dimer and in subjects with a high clinical probability of disease. CUS can be negative in the case of pelvic vein thrombosis and can have a non optimal sensitivity in symptomatic subjects with distal DVT [28].

### Ultrasound characterization of lung consolidations

When the air spaces of the lung are substituted and filled with liquid, semiliquid or solid (exudate, proteinaceous and fibrinous material, pus, blood or neoformative tissue) material, “hepatization” occurs from the anatomo-pathologic point of view, which corresponds from the radiological point of view to radiopaque lung consolidation [4].

Lung consolidation in view of its characteristics is suitable for a classically morphological lung ultrasound assessment only if it emerges at pleural level. Only in this case, in fact, does the presence of lung consolidation reduce the high difference of acoustic impedance existing at the pleural line between ventilated lung and chest wall and permit the penetration of ultrasounds.

Lung consolidation can be of diverse nature: atelectatic, inflammatory (pneumonia), infarctual, contusive, or heterogeneous.

Whatever its nature, the possibility of carrying out a morphological examination allows the investigation of some sonographic characteristics of lung consolidations that may be useful for their etiologic definition. One of the most important signs to look for is the bronchogram [29]. There are two distinct types of bronchogram: air and fluid.

The air bronchogram sign sonographically appears as hyperechogenic foci, spots and stria. It can be dynamic or static according to the possibility of visualizing the movement of air in the bronchi consensually with the act of breathing. Sonographic air bronchogram is dynamic and appears irregular and with sometimes a typically tree-like form in lung consolidations of an inflammatory nature. In atelectasis, especially if obstructive, it can be completely absent; if present, it appears instead static and with a more regular, parallel course. It is often associated with the “lung pulse” sign [30], resulting from the perception of cardiac activity at pleural line level when the sliding sign is only very slight or completely absent.

Sonographic fluid bronchograms, on the other hand, appear within a lung consolidation as tubular structures with a hypo- or anechoic content, without any flow at Doppler sampling and hence distinguishable from blood vessels [4]. These structures represent bronchi with fluid content and are indicative of post-obstructive pneumonia.

Use of ultrasound contrast agents administered intravenously (phospholipid-stabilized microbubbles filled with sulfur hexafluoride, SonoVue®, Bracco) can be of further help in the etiopathologic definition of pleural-based lung consolidation. The sonographic characteristics of lung consolidations investigated through use of a contrast medium are described on the basis of time (“time to enhancement”, TE) and extent (“extent of enhancement”, EE) [31]. TE corresponds to the time it takes between the intravenous infusion of the means of contrast and the enhancement of the lung consolidation. If the appearance of the contrast enhancement (CE) occurs within 6 seconds the TE is defined “early” and it indicates a pulmonary artery vascularization. If the TE appears after 6 seconds it is defined “late” and it indicates a systemic artery vascularization (bronchial arteries).

EE, on the other hand, is assessed in relation to the enhancement of the parenchymatous tissue of the spleen. It can be “marked” or “reduced”.

Further distinguishing characteristics are based on the homogeneity or less of the impregnation with means of contrast and on the disposition of the vascular structure in the sample of the Doppler examination. One can distinguish, then, the following pictures which can be of real help in determining the nature of the lung consolidation:

1) Inflammatory consolidation: early TE and marked EE. CE homogeneous. In small subpleural inflammatory lesions the CE can be dishomogeneous in the pulmonary arterial (early) phase. Vascularization with non altered vascular architecture.

2) Consolidation associated to pulmonary embolism: a late TE and reduced EE can be observed. CE dishomogeneous, particularly in the initial phases of impregnation. In some cases also the late enhancement can be missing. In patients with septic embolism the lesion can manifest a central area completely and persistently without contrast.

3) Atelectasis: early TE and marked EE. CE homogeneous right from the pulmonary arterial phase. Vascularization with non altered vascular architecture. Especially if the cause is compressive, one observes a delayed wash out of the contrast in relation to the other parenchymatous organs such as liver and spleen.

4) Neoplasm: late TE and variable EE. CE dishomogeneous often with progression from the periphery to the central zones. The vascularization appears altered, diffuse within the lesion with “muff-like” areas and focal areas. Necrotic areas that do not present CE may be visualized.

### Ultrasound-guided interventional procedures

In daily clinical practice, lung ultrasound can – or “better” should – be used also as a guide in interventional procedures in pulmonology such as thoracentesis, tube insertion for chest drainage, transcutaneous biopsies of the pleura and peripheral lung parenchyma, thoracoscopy.

#### Thoracentesis

Regarding use of lung ultrasound for performing thoracentesis and/or insertion of chest drainage tubes, with the aid of ultrasound one can identify the best site for the procedure, taking into account the need for sufficient depth of pleural fluid (at least 10 mm is considered safe) and the absence of lung parenchyma interposed (the assessment should be carried out, if possible, with maximal inspiratory maneuvers). One can thus proceed with the so-called “X marks the spot” technique, identifying by ultrasound the best site for the thoracentesis/drainage tube insertion and then executing the procedure guided by the preceding ultrasound evaluation, or else one can perform the procedure under constant sonographic visualization, taking advantage of all its benefits/advantages [32].

The use of lung ultrasound as a guide for procedural interventions is particularly useful in critical care conditions, given the combination of a relatively immobile patient and the feasibility of “bedside” performance of the procedure. The possibility of identifying and guiding with security the aspiration of pleural effusions in patients on mechanical ventilation is well known [33]. The advantages of lung ultrasound-guided thoracentesis as regards rates of pneumothorax following thoracentesis were recently spelt out in the first systematic review and meta-analysis conducted on articles published between January 1st 1996 and 1st April 2009. This study documented the overall rate of pneumothorax-complicated thoracentesis at 6%, with requirement of chest drainage tube insertion in 34.1% of cases (1.7% of all thoracenteses performed). The possibility of instant recourse to ultrasound-guided procedures has been associated to a significantly lower risk of pneumothorax [34].

#### Chest drainage tube insertion

Evidently these same premises are also valid for positioning of the drainage tube in its most varied indications. Chest drainage systems now available are smaller, more precise, atraumatic, equipped with guide-wires, adapted to the most disparate situations and well tolerated. Major international guidelines have for long recognized the absolute utility of ultrasound as a guide, proposing it as indispensable [35]. Inserting a tube without such aid is to be considered incorrect; the risks of malpositioning and of lesions to the vital organs of chest and subdiaphragm are practically absent with ultrasound guidance, and the efficacy extremely high. Ultrasound is fundamental in preparing the placement not only of small catheters (on which a substantial literature exists [36,37]) but also of the larger trocars (24-28F). And in infectious effusions, notoriously “difficult-to-manage” and multi-loculated, hidden at times between the folds of parenchyma adhering to the wall, a sonographic approach is absolutely fundamental: it permits the drainage of the more voluminous loculations and indicates the successive therapeutic strategy, e.g. instillation of fibrinolytic agents or recourse to evacuative injections in the case of non-drained loculations. In all this ultrasound has proven to be superior to CT scan which is notoriously incapable of seeing the sepimentations within such effusions. It should also not be forgotten that with high-frequency linear probes and Doppler it is possible to identify with a good approximation the course of the intercostal arteries and veins, thus avoiding their perforation or, worse still, their laceration during the insertion [38]. A final point to underline is that ultrasound gives the possibility, once the tube has been positioned, of following the drainage process within the pleural cavity [39].

#### Transcutaneous biopsies of the pleura and lung parenchyma

There are three targets in the chest that can be approached correctly and efficaciously with ultrasound guidance: chest wall lesions in all their thickness, pleural lesions of any nature occupying space from nodules to areas of thickening, and peripheral pulmonary lesions in contact with the wall can be biopsied under the constant, attentive control of ultrasound. The approach is simple and precise, real-time, quick and without substantial risks [40]. Various needles can be used, from the simple Menghini sharp-pointed needles (21–18 G) to the more “aggressive” cutters of various caliber (14-18-21G) that allow frustules to be extracted suitable for histologic examination. The coupling of needle and probe enables a constant view of the needle as it reaches its target, once the lesion has been defined in terms of its size, contour, echogenicity and vascularization [41]. The easiest technique is that of applying to the probe lateral adaptors that block the needle and, the angle of entry being predefined, direct it to the target through a fixed course on the screen. This approach does not require particular experience but it is limited by the fact that often (given that the relation between needle and probe is fixed and narrow and the rib cage protected by the ribs) it can be impossible to reach the lesion due to rigidity of the whole apparatus. More difficult but, once learned, far more effective is the “free hand” technique, whereby small adjustments of needle inclination and consensual adjustments of the probe permit almost always a good success of the maneuver [42]. Speed is the true advantage of the ultrasound-guided approach to transthoracic biopsy. The efficacy is similar to that with the other imaging techniques, CT in first place, but the time that the needle spends in the lesion is far less with ultrasound, with a gain in tolerability and less complications. Ultrasonography, finally, permits to identify eventual post-procedural complications such as pneumothorax. The diagnostic yield, in expert hands, exceeds 90% [43].

#### Thoracoscopy

Thoracoscopy is now fully accepted as part of the daily practice of respiratory medicine and its field of application and indications are constantly increasing [44]. The classical methodology involves a preparatory pneumothorax induced generally with blunt needles the day before or immediately prior to the examination. Ultrasonography can easily substitute this practice rendering the examination more productive and safer. Ultrasounds in addition provide other preliminary information that is useful in the course of endoscopy such as the involvement of the diaphragm, the presence of masses and/or adherences to be avoided during insertion of the trocar, the status of the lung parenchyma, the echogenicity of the material present, and the sepimentation of the same [45]. But where sonography is really superlative is in those situations where it is necessary to examine the pleural surface in the absence of liquid. An attentive analysis of the lung’s sliding by means of a careful and extended mapping of the “sliding sign” provides precious information on the lung’s collapsibility and on whether an effective endoscopic recognition is possible. If present, the “sliding” sign authorizes a prudent entry by blunt curve with introduction of the stylet (generally of 7 mm diameter in medical thoracoscopy) without producing damage and with immediate formation of a pleural chamber suitable for the exploration and for the successive biopsies [46-48].

Further, ultrasound after the examination allows to monitor the pulmonary re-expansion as well as to control in infectious effusions the course of the residual loculations when the thoracoscopy toilet has not been complete, and any eventual malpositioning of the drainage. Also after pleurodesis a good ultrasound examination can provide precious information about its efficacy and maintenance over time [49].

### Lung ultrasound in neonatal and pediatric care

Newborn and young infants show at ultrasound exploration of the lung similar pictures to those of adults. The exploration uses high-frequency linear probes (7.5-12 MHz) executing longitudinal and transverse scans in the anterior, lateral and posterior regions of the chest.

It is well known that early exposure to ionizing radiation of neonatal and young infants involves an elevated risk of developing neoplasms in the course of the years. Ultrasound avoids this risk.

Despite this, at the current stage, the use of ultrasounds for examination of the lung on the part of neonatologists and pediatricians is still not widespread. This writer has the conviction that over and above its diagnostic validity, the use of ultrasounds in these age-groups responds also to a problem of professional ethics [50].

#### Technique of execution and sonographic anatomy

The examination is carried out with the infant in supine position. The probe is placed perpendicularly on the skin and longitudinal, transverse and oblique scans are made in the anterior, lateral and posterior chest regions. The posterior areas can be assessed in lateral decubitus position. Sitting position is almost never necessary, especially in the newborn. Normal neonatal lung does not substantially differ from that of the adult. The pleural line is easily identifiable below the rib level as also is the “sliding sign” [51].

The presence of horizontal reverberations of the pleural line (A lines) identifies a condition of normality [51].

At birth it is possible to visualize vertical artifacts (B lines), pathognomonic of adult sonographic interstitial syndrome [1,2,14,51], also in completely healthy neonates [52]. The fetal lung is in fact very rich in water and thus B lines can be present in healthy neonates, more frequently if born by cesarean delivery [52]. In good part this is due to the absence of compression of the rib cage along the birth canal. B lines in these circumstances are more numerous on the right without, though, a typical localization and tend to disappear completely within a period of 24–36 hours.

#### Transient tachypnea of the newborn (TTN)

TTN is a frequent cause of neonatal respiratory distress. It has a low morbidity but if severe poses problems of differential diagnostics with other pulmonary or cardiac pathologies (e.g. pneumothorax, pneumonia, sepsis, hyaline membrane disease, congenital heart disease).

All newborn infants with TTN evidence at the lung base, bilaterally, a picture of coalescent B lines (“white lung”) and a normal or pseudo-normal aspect (presence of non coalescent B lines) in the upper fields [52]. The limit between the lower fields (“white lung”) and upper fields of the lung is so clear that the ultrasound picture results absolutely specific. It is important to stress that the pleural line appears normal also in the areas of “white lung”. This peculiar sonographic finding has been called “double lung point” in that one seems to observe two different lungs side by side in the same patient.

#### Hyaline membrane disease (HMD)

HMD is, at least in part, due to the deficit of surfactant and concerns in particular premature babies.

All affected neonates evidence a picture of bilateral “white lung”. The pleural line is always involved with a thickened, irregular, poorly definable appearance. Multiple hypoechogenic areas, of small dimensions, are observable particularly in the posterior and lateral fields and are an expression of areas of lung consolidation. Consolidations of large dimensions with evidence of fluid bronchograms can be observed always in the posterior fields.

These findings are present at birth and precede the newborn’s clinical deterioration [53].

In summary, there are three fundamental ultrasound signs: 1) bilateral “white lung”, 2) absence of “spared areas” (areas of normal lung), 3) alterations of the pleural line.

#### Bronchopulmonary dysplasia (BPD)

BPD is a form of chronic pulmonary disease that interests premature neonates who are on oxygen therapy and mechanical ventilation. BPD manifests ultrasonographically with the presence of multiple B lines that present a non homogeneous distribution and with diffuse alterations of the pleural line (thickening, irregularity and presence of multiple small subpleural consolidations).

Generally “spared areas” are present, and the extent of the interstitial syndrome and pleural line alterations are correlated to the severity of the clinical picture [53].

#### Pulmonary atelectasis

Pulmonary atelectasis is frequent in ventilated neonates. Often the X-ray diagnosis is difficult on account of the underlying pulmonary disease. The dynamic ultrasound signs can be extremely useful and monitored at the patient’s bedside. The ultrasound aspect of atelectasis, as in the adult, is characterized by an area of consolidation, by “lung pulse” [30], by the absence of sliding and by the parallel course of the bronchi. The evidence of dynamic air bronchograms excludes obstructive atelectasis [29].

These elements are important in that often pulmonary consolidations can be secondary to alveolar collapse (e.g. lung hemorrhage, HMD) and in ventilated newborn infants to hypoventilation.

#### Pneumothorax

Pneumothorax is frequent in the newborn. Chest X-ray presents the same limits well known in the adult. Transillumination is the diagnostic technique most used by neonatologists. Sonographic signs are the same as those described in the adult: 1) absence of sliding, 2) absence of B lines, and 3) presence of “lung point” in non massive pneumothoraxes [3].

#### Bronchiolitis

Bronchiolitis is an acute airways infection that can involve obstruction of the bronchioles. Diagnosis is based on seasonal recurrence and on the clinical picture. The ultrasound findings are peculiar and this is important due to the fact that in the most severe cases lung ultrasound can avoid the need for a chest X-ray.

Individuals affected by bronchiolitis always manifest a bilateral lung involvement characterized by interstitial syndrome that is dishomogeneous in terms of its extent and distribution, associated to the presence of subpleural consolidations which are generally of small size. Extensive consolidations can be observed in the more severe forms. Small pleural effusions are frequent.

Recently Caiulo et al. [54] showed that the use of ultrasound can drastically reduce the need for chest X-ray. Finally, observations as yet unpublished of the group of Basile and Comes (Monopoli-Bari) have shown an early appearance of the interstitial syndrome in the paravertebral areas.

#### Pneumonia

Infants affected by pneumonia can present a classical picture at clinical observation or also appear without respiratory signs and symptoms. Chest x-ray is still considered the image diagnostics to use as a first step for the diagnosis. In infants pneumonia appears at ultrasound as a hypoechogenic area with a poorly defined contour, frequently surrounded by B lines. The pleural line in the vicinity of the consolidation appears hypoechogenic and with reduced or absent sliding. In the case of extensive consolidations one can observe tree-form hyperechogenic structures, an expression of air bronchograms, in the vicinity.

Dynamic air bronchograms can be present, in which case obstructive atelectasis can be immediately excluded. Multiple hyperechogenic spots, expression of air trapped in the bronchioles, are also frequently present. Fluid bronchograms, frequently described in post-obstructive pneumonias, are frequent in infants and appear as tubular structures with hyperechogenic walls with anechoic content. Pleural effusions are easily identifiable and appear as anechoic areas in the pleural space. Of particular use, as in the adult, is the possibility of being able to identify early the development of effusions into empyema [55].

In pediatric age, similarly as in the adult, lung ultrasound has demonstrated a superior diagnostic accuracy, in any case not inferior with respect to chest X-ray [56].

## Conclusions

Chest ultrasonography can be a useful diagnostic tool for respiratory physicians to assess and monitor respiratory pathologies in many different conditions with wide field of application.

In this document II it is shown how chest ultrasonography can help physicians to get differential diagnosis in sonographic interstitial syndromes and acute cardiogenic/non-cardiogenic pulmonary edema, trying to give advanced hypotheses about the genesis of vertical artifacts.

Moreover, it is shown its utility to raise diagnostic suspicion of pulmonary embolism, to characterize lung consolidations and to guide interventional procedures in pulmonology.

Finally it is shown how chest ultrasonography may be a useful diagnostic tool in pediatric and neonatal care and could be used in many different pathological conditions avoiding ionizing radiations.

## Competing interests

The authors declare that they have no competing interests.

## Authors’ contributions

AS, RI, GS, RC, GM, AZ, RG, AT, SN, SV: all authors contributed to draft and to write the manuscripts, to review them and to collect articles and references. All authors are responsible of the whole manuscript, both document I and document II.

AS, RI, GS, RC, GM, SV in particular cured the final drafting of the Document II AS, AZ, RG, AT, SN in particular cured the final drafting of the Document I. All authors read and approved the final manuscript.
